# Collapsin response mediator proteins: Potential diagnostic and prognostic biomarkers in cancers (Review)

**DOI:** 10.3892/ol.2014.1909

**Published:** 2014-02-24

**Authors:** FEI TAN, CAROL J. THIELE, ZHIJIE LI

**Affiliations:** 1 Department of Neurology, Shengjing Hospital of China Medical University, Shenyang, Liaoning 110004, P.R. China; 2 Cell and Molecular Biology Section, Pediatric Oncology Branch, National Cancer Institute, National Institutes of Health, Bethesda, MD 20892, USA; 3 Research Center for Medicine, Shengjing Hospital of China Medical University, Shenyang, Liaoning 110004, P.R. China

**Keywords:** collapsin response mediator proteins, biomarker, tumor suppressor, metastasis

## Abstract

The collapsin response mediator proteins (CRMPs) were originally identified as mediators of semaphorin 3A signaling and neuronal differentiation. The CRMP family consists of five homologous cytosolic proteins, CRMP1-5. Altered expression levels of CRMPs have been observed in several malignant tumors, including lung, breast, colorectal, prostate, pancreatic and neuroendocrine lung cancer. The aim of the current study was to review the recent progress achieved in understanding the association between the different levels of CRMP expression in tumors and their involvement in pathological functions, such as tumor metastasis, disease progression, subtype differentiation and clinical outcome, to address the potential value of CRMPs as biomarkers for the diagnosis and prognosis of cancer patients.

## 1. Introduction

Collapsin response mediator proteins (CRMPs) are cytosolic phosphoproteins that are highly expressed in the developing and adult nervous system. CRMPs were originally identified by different authors and, thus, have been termed as turned on after division, 64 kDa protein (TOAD-64) (1), dihydropyrimidinase-related protein (DRP) (2), uncoordinated 33-like protein (Ulip) (3), TOAD-64/Ulip/CRMP (TUC) (4) and CRMPs (4). This group of proteins is also known as the dihydropyrimidinase-like protein (DPYSL) family. However, the most frequently used name in the medical literature is CRMP and, therefore, has been adopted in the present review. The CRMP family consists of five homologous cytosolic proteins that are expressed in distinct yet partially overlapping patterns of expression during the development of the nervous system in humans, rats and mice (5–8). The nomenclatures of the five members of the CRMP family, which are used in different fields of study, are listed in Table I.

CRMPs are the mammalian homologues of the *Caenorhabditis elegans* Ulip gene. Mutations in the unc-33 gene result in severely uncoordinated movement, abnormalities in the guidance and outgrowth of the axons of a number of neurons and a superabundance of microtubules in neuronal processes (9). Although CRMPs were originally identified as mediators of semaphorin 3A (Sema3A)-induced growth cone collapse, the significant similarity between the CRMP and worm unc-33 gene sequences indicates an additional role for CRMPs in the growth and morphology of axons. Previous studies have indicated that in cultured neurons CRMPs have a multifunctional role in neuronal development, including axon formation/extension (CRMP1) (10), axonal guidance, specification, elongation and branching (CRMP2 and CRMP4) (11–13), filopodial dynamics and growth cone development (CRMP5) (14).

**Figure 1:**
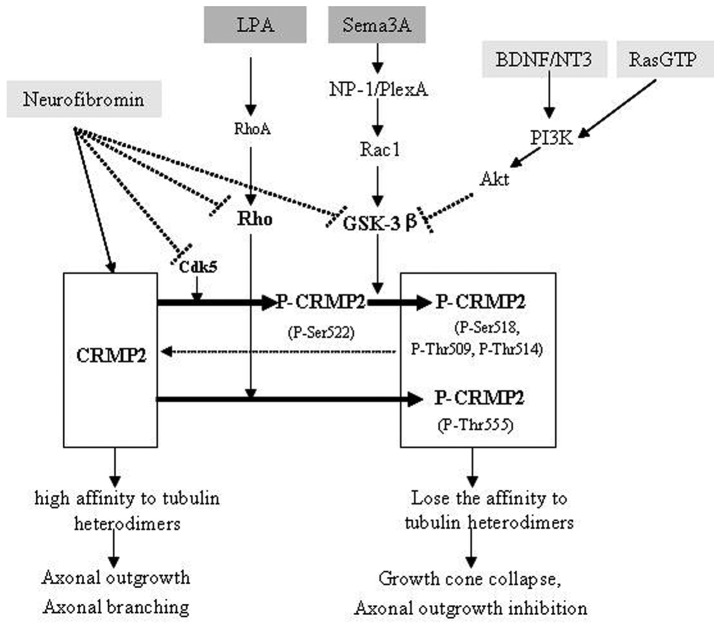
Signaling pathways regulating growth cone collapse, axonal outgrowth and axonal branching through CRMP2. Sema3A triggers Rac1 activation, via its receptors NP-1 and PlexA1. This is followed by the activation of GSK-3β which subsequently phosphorylates CRMP2 at Ser-518, Thr-509 and Thr-514. The GSK-3β-mediated CRMP2 phosphorylation is dependent on the prior phosphorylation of CRMP2 at Ser-522 by Cdk-5. LPA induces CRMP2 phosphorylation at Thr-555 via RhoA and Rho, and BDNF, NT3, and Ras-GTP inhibit the activation of GSK-3β via PI3K and Akt to activate CRMP2. Neurofibromin directly interacts with or activates CRMP2 by inhibiting Cdk5, Rho or GSK-3β. Phosphorylated CRMP2 loses it affinity to tubulin heterodimers and subsequently induces growth cone collapse or axonal outgrowth inhibition. CRMP2, collapsin response mediator protein 2; Sema3A, semaphorin 3A; Rac1, Ras-related C3 botulinum toxin substrate 1; NP-1, neuropilin-1; PlexA1, plexin A1; GSK-3β, glycogen synthase kinase 3β; Cdk5, cyclin dependent kinase-5; LPA, lysophosphatidic acid; RhoA, Ras homolog family member A; BDNF, brain-derived neurotrophic factor; NT3, neurotrophin 3; PI3K, phopshoinositide 3-kinase; Akt, protein kinase B.

Among the five CRMP family members, CRMP2 was the first to be identified; therefore, the majority of studies have analyzed the mechanisms of CRMP2. In Fig. 1, the signaling pathways that regulate CRMP2 expression and its associated biological functions are summarized.

CRMP2 was originally identified as the signaling molecule of the repulsive guidance cue, Sema3A, which induces growth cone collapse (15). Sema3A triggers Ras-related C3 botulinum toxin substrate 1 (Rac1) activation through its receptors neuropilin-1 (NP-1) and plexin A (PlexA), which affects the downstream kinases and subsequently leads to the activation of glycogen synthase kinase 3β (GSK-3β). GSK-3β then phosphorylates CRMP2 at Thr-509, Thr-514 and Ser-518. However, the phosphorylation of CRMP2 at Ser-522 by cyclin dependent kinase-5 (Cdk5) is a prerequisite for GSK-3β-mediated CRMP2 phosphorylation. The phosphorylated CRMP2 loses affinity for tubulin heterodimers, which induces tubulin depolymerization and microtubule destabilization, thus reducing microtubule growth at the distal end of axons to encourage axon retraction (11,16–18). The phosphorylation at Ser-522 by Cdk5 and the subsequent phosphorylation at Thr-509, Thr-514 and Ser-518 in CRMP1 and CRMP4 was identified to be similar to that in CRMP2. By contrast, Ras (19), neurotrophin-3 (NT-3) (20) and brain-derived neurotrophic factor (BDNF) inhibit GSK-3β via the phosphatidylinositol-3-kinase (PI3K)/Akt pathway, which subsequently reduces CRMP2 phosphorylation (pThr-509/pThr-514) and promotes axon growth (19–21).

An additional signaling pathway that causes neuronal growth cone collapse, but is Sema3A-independent, is lysophosphatidic acid (LPA) signaling (22,23). LPA is a bioactive phospholipid and during LPA-induced growth cone collapse, CRMP2 is phosphorylated at Thr-555 by Rho-kinase, which is downstream of RhoA (24). CRMP2 mediates Sema3A signaling, which is independent of the phosphorylation by Rho-kinase (24).

Neurofibromin is the neurofibromatosis type 1 tumor suppressor gene product. Previous studies have identified a novel function of neurofibromin in neurite outgrowth, involving the regulation of CRMP2 phosphorylation via direct and indirect associations with CRMP2. Neurofibromin directly interacts with the active (non-phosphorylated) form of CRMP2 via the neurofibromin c-terminal domain, but does not interact with the inactive (phosphorylated) form of CRMP2. Neurofibromin and CRMP2 are particularly colocalized in the distal tips and branches along the neuritis, which are recognized as major CRMP2 active sites in the differentiated PC12 cells. The downregulation of neurofibromin induces the inactivation of CRMP2 through phosphorylation regulated by Cdk5, GSK-3β and Rho-kinase, which induces the neurite retraction of the cells (25). Therefore, the functional association between neurofibromin and CRMP2 is essential for neuronal cell differentiation.

Although CRMPs were originally identified in the nervous system and are involved in neuronal development, studies have demonstrated that CRMPs are expressed in cancerous tissues and may have an effect on cancer progression and metastasis.

## 2. CRMPs as biomarkers in cancer

### CRMP1 and lung cancer

Similarly to CRMP2, CRMP1 is also a mediator of extracellular guidance cues, such as Sema3A, and contributes to cytoskeletal reorganization in the axonal pathfinding process. The involvement of CRMP1 in cancer progression was first reported by Shih *et al* (26)who compared poorly and highly metastatic lung cancer cells using a cDNA microarray, whereby metastasis-associated genes were identified on a genome-wide scale. Cluster analysis of the cDNA microarray results revealed that ~500 genes were positively or negatively correlated with cancer cell invasiveness; the majority of these genes were involved in angiogenesis, cell motility, adhesion and proliferation. However, the expression of CRMP1 mRNA was identified to negatively correlate with cell invasiveness (27). The association between an invasive phenotype and CRMP1 expression *in vitro* was evaluated by genetic manipulation of CRMP1 expression. Transfection of a CRMP1 construct into a highly invasive lung cancer cell line was found to significantly reduce the invasive activity of the cells, whereby morphological changes (from an elongated to a rounded morphology) and a reduction in filopodia were observed. A previous study evaluated the association between CRMP1 expression in tumors and clinical outcome in 80 lung cancer patients. The results indicted that the expression of CRMP1 mRNA in non-small cell lung cancer (NSCLC) was significantly lower than that in the adjacent normal tissues. The median value of CRMP1 mRNA expression was used to classify patients into high or low expression groups, whereby patients with low CRMP1 expression were found to exhibit a more advanced level of disease and lymph node metastasis. By contrast, the high expression group exhibited a significantly longer disease-free and overall survival than those of the low expression group (26, 28). This study implicated CRMP1 as a novel invasion suppressor gene in lung cancer.

Cyclooxygenase-2 (COX-2) is considered to be important in the regulation of CRMP1 in lung cancer; the overexpression of COX-2 has been reported to decrease CRMP1 mRNA and protein expression. By contrast, the COX-2 inhibitor, celecoxib, was found to induce a dose-dependent increase in CRMP1 expression. COX-2 inhibitors increase CRMP1 expression by inhibiting Sp1-DNA complex formation and enhancing the DNA binding of the CCAAT-enhancer-binding protein α at the promoter (29). Previous studies have identified that COX-2 is overexpressed in lung cancer and that COX-2 expression promotes lung cancer cell proliferation, invasion and angiogenesis (30, 31). COX-2 inhibitors suppress cancer development by inducing apoptosis or inhibiting prostaglandin synthesis, cell cycle progression, angiogenesis or metastasis (32–34). Therefore, COX-2 may increase metastasis by the regulation of CRMP1 expression in lung cancer cells. Additionally, connective tissue growth factor (CTGF), a secreted protein that binds to integrins on the cell surface, has been observed in breast cancer, pancreatic cancer, melanoma and chondrosarcoma (35–38). The expression of CTGF has been found to be associated with anti-invasive and antimetastatic activities in human lung adenocarcinoma. In addition, reduced CTGF expression in tumor tissues has been associated with advanced tumor stage, lymph node metastasis, early postoperative relapse and shorter patient survival (39). This study indicated that CRMP1 acts as a downstream effector of CTGF and mediates the anti-invasive and antimetastatic activities of CTGF (39).

Pan *et al* (40) identified a novel human isoform of the CRMP family proteins termed long-form CRMP1 (LCRMP1), which can be distinguished from the known invasion suppressor, CRMP1, by its molecular weight and N-terminal exon 1. The authors revealed a positive correlation between LCRMP1 expression and lung cancer cell invasiveness. Additionally, high LCRMP1 mRNA expression was associated with poor overall and disease-free survival in NSCLC patients. The metastatic lymph nodes demonstrated higher LCRMP1 expression when compared with that of the primary lung tumors. Therefore, LCRMP1 was considered to be a cancer invasion enhancer, which may present as a novel prognostic biomarker in NSCLC (41). To further understand the mechanisms associated with the cancer cell migration and invasiveness of LCRMP1, Pan *et al* (40) also revealed that the overexpression of LCRMP1 in non-invasive human cell lines enhances filopodia formation and cancer cell migration and invasion via the stabilization of actin through binding to the Wishott-Aldrich syndrome protein family 1 (WAVE-1) protein. CRMP1 and LCRMP1 were found to directly interact with each other to form heterodimers in cells, however, CRMP1 did not interact with WAVE-1. In addition, the overexpression of CRMP1 was found to inhibit the interaction between WAVE-1 and LCRMP1, which resulted in the regression of filopodia and suppression of the invasive ability in LCRMP1-overexpressing cells. Such observations suggest that CRMP1 and WAVE1 may compete for LCRMP1 binding, which may highlight the mechanism through which these proteins affect filopodia formation and cancer cell invasion/migration (41).

A study comparing CRMP1 and LCRMP1 expression levels in tumor specimens obtained from 142 patients with NSCLC indicated that patients with low levels of CRMP1 expression or high levels of LCRMP1 expression had a poorer overall and disease-free survival. CRMP1 and LCRMP1 are independent prognostic factors and analysis of the combined effect of the two proteins on patient prognosis has revealed that patients with high levels of CMRP1 expression and low levels of LCRMP1 expression demonstrate an improved overall and disease-free survival than patients exhibiting low levels of CRMP1 expression and high levels of LCRMP1 expression. These results indicate that CRMP1 and LCRMP1 counter-regulate cancer metastasis in the clinic (41). LCRMP1 and CRMP1 exhibit opposing functions in the regulation of cancer cell invasion and metastasis, which may implicate this pathway as a potential anticancer target.

In addition, LCRMP1 appears to act downstream of cell division control protein 42, a Rho family protein which is involved in actin rearrangement (42). GSK-3β has been observed to phosphorylate LCRMP1 at Thr-628 and Ser-632 in lung cancer cells, and the Thr-628 of LCRMP1 has been identified as a dominant and important phosphorylation site for LCRMP1 function. The GSK-3β-dependent phosphorylation of LCRMP1 positively regulates filopodia formation, migration and cancer cell invasion. Furthermore, clinical NSCLC patients with low levels of inactive GSK-3β and high levels of LCRMP1 protein expression demonstrated a poorer overall survival than patients with high levels of the inactive form of GSK-3β and low levels of LCRMP1 expression (42). These results suggest that high activity of GSK-3β and high levels of LCRMP-1 expression, are associated with increased cancer invasiveness and poorer overall survival in NSCLC patients. Therefore, a new regulatory mechanism for GSK-3β has been highlighted which involves the phosphorylation of the invasive enhancer LCRMP1.

### CRMP2 and cancer

Although the majority of studies have focused upon the involvement of CRMP2 in neuronal development, its function in tumorigenesis has also been investigated. Wu *et al* (43) analyzed the secretomes of 21 cancer cell lines derived from 12 types of colorectal carcinoma and revealed the detection of CRMP2 in the colorectal carcinoma cell line secretome. Furthermore, CRMP2 mRNA expression was detected in 43 colon cancer tissues. CRMP2 was identified as the major CRMP isoform expressed in normal and tumorous colon tissues, and CRMP2 expression levels were found to be significantly higher in tumor tissues than normal tissues, however, no significant differences in expression were observed in the four other CRMP family members. The CRMP2 protein expression was investigated in 169 colorectal carcinoma tissue sections and the results revealed the positive staining of CRMP2 in 58.6% of the tumors; however, in 90% of the adjacent non-tumor epithelial cells, weak or no expression of CRMP2 was observed. Furthermore, CRMP2 was predominantly located in the cytoplasm of tumor cells. The percentage of positive CRMP2 staining was identified to be significantly higher in the early stage of the disease compared with the late stage, and significantly higher in early lymph node metastasis. However, CRMP2 expression was not found to significantly correlate with tumor metastasis stage or five-year survival, although, patients with CRMP2-positive tumors tended to demonstrate improved five-year survival. The plasma CRMP2 levels were detected to investigate its role as a biomarker in colorectal carcinoma. The results indicated a significantly higher levels of CRMP2 expression in colorectal carcinoma patients when compared with those of the healthy controls; however, CRMP2 plasma levels in colorectal carcinoma patients were not found to significantly correlate with metastasis or lymph node metastasis (43). These results indicated that CRMP2 may be suitable as a biomarker for the diagnosis of colorectal carcinoma, but not as a prognostic indicator.

An additional study by Oliemuller *et al* investigated the protein expression of CRMP2 in 91 NSCLC patients (44). The authors identified that CRMP2 was expressed in the cytoplasm of normal and tumor tissues, with a higher expression exhibited in tumor tissues than in normal tissues. Phosphorylated CRMP2 was also investigated and identified only in the tumor tissue, predominantly located in the nucleus. The authors analyzed the association between CRMP2 and clinical outcome in NSCLC patients and revealed that total CRMP2 expression was not associated with overall survival or recurrence-free survival. Such results are similar to those described in the colorectal carcinoma study by Wu *et al* (43). While, a significant correlation was identified between the high levels of phosphorylated CRMP2 (Thr-514) and shorter overall survival in the study by Oliemuller *et al* (44). However, the correlation between high levels of phosphorylated CRMP2 expression and poor overall survival was only demonstrated in patients who did not receive adjuvant therapy. These results indicate that the phosphorylated form of CRMP2 may present as a prognostic marker for NSCLC. The mechanism of this observation was investigated, which revealed that CRMP2 is involved in cell division in a phosphorylation-dependent manner. CRMP2 is phosphorylated at the Thr-509, Thr-514 and Ser-518 residues by the protein kinase, GSK-3β. CRMP2 phosphorylation impairs its binding to tubulin and delays spindle assembly and entry into the metaphase of the cell cycle. By contrast, non-phosphorylated CRMP2 increases microtubule stabilization and interferes with the cell cycle progression into anaphase. Additionally, overexpression of phospho-defective mutant CRMP2 increases the expression of P53 and P21, which induce apoptosis. Therefore, the presence of phosphorylated forms of the CRMP2 protein appear to be a distinctive feature of highly proliferative cells. Thus, in NSCLC, CRMP2 expression is crucial for cell survival, and may be a useful therapeutic target (44).

The expression of CRMP2 in breast cancer was previously investigated by Shimada *et al* (45), who revealed that the mRNA and protein expression of CRMP2 were significantly decreased in breast cancer tissues compared with that in the normal tissues. However, no statistically significant difference was identified in the expression of the other CRMP family members (CRMP1 and CRMP3-5). Additionally, in breast cancer tissues, CRMP2 expression was not found to significantly correlate with histology, tumor diameter, histological grade, lymph node metastasis, lymphatic or venous invasion, or tumor stage. However, the expression of phosphorylated CRMP2 was detected in breast cancer tissues, but not in normal mammary tissues. Furthermore, the phosphorylated CRMP2 was found to predominately localize in the nuclei of breast cancer cells and was found to significantly increase in tumors with a higher histological grade (45). The phosphorylation of CRMPs is sequentially catalyzed by two kinases, Cdk5 and GSK-3β. Cdk5 has been previously shown to be involved in the proliferation of invasive breast cancer cells, via the antagonization by the Cdk inhibitor, roscovitine, or by targeted knockdown of Cdk5 (46,47). The increased nuclear localization of GSK-3β in breast cancer tissues has also been reported (48). Thus, the phosphorylation of CRMP2 in the nuclei of breast epithelium by Cdk5 and GSK-3β may contribute to breast cancer progression.

The aforementioned results indicated that, although total CRMP2 levels were differentially expressed in various tumors (increased expression in NSCLC and colorectal carcinoma tissue, and decreased expression in breast cancer) and had no association with clinical outcome, a common change in the levels of phosphorylated CRMP2 was observed in NSCLC and breast cancer. In the two tumor types, phosphorylated CRMP2 was detected in the nuclei which was found to be associated with the clinical outcome of patients. Therefore, phosphorylated CRMP2 may present as a prognostic biomarker in NSCLC and breast cancer, and possibly other tumors.

### CRMP4 and cancer

Prostate cancer is the most common type of solid organ malignancy affecting males. Among the adverse pathological features, the presence of pelvic lymph node metastasis is the strongest predictor of poor outcome. Using proteomic-based expression profiling, Gao *et al* (49) identified that CRMP4 was significantly decreased in pelvic lymph node metastatic prostate cancer compared with localized prostate cancer. Western blot analysis and real-time polymerase chain reaction (RT-PCR) procedures verified the proteomic results. CRMP4 expression was detected predominantly in the cytoplasm of epithelial cells and its expression was evident in all the benign prostate hyperplasia specimens observed. The authors also analyzed the expression patterns of all five members of the CRMP gene family (CRMP1-5) in benign prostate hyperplasia and prostate cancer tissues. However, of all the CRMP family members, the CRMP4 gene was determined to be the only gene differentially expressed in prostate cancer tissues.

Analysis of the correlation between CRMP4 expression and postoperative relapse and clinical metastasis identified that patients with localized prostate cancers, with a relatively low CRMP4 expression level at diagnosis, exhibited rapid biochemical relapse and clinical metastasis. Furthermore, the expression levels of CRMP4 were found to decrease in these patients with progression from localized to metastatic lesions. However, patients with lymph node metastatic prostate cancer with high CRMP4 expression levels demonstrated no biochemical relapse or clinical metastatic findings until the two-year follow-up. These data implicate CRMP4 as a metastasis suppressor and progression predictor in prostate cancer. To investigate the mechanism by which CRMP4 expression is downregulated in metastatic prostate cancer, Gao *et al* (49) analyzed the structure of the CRMP4 promoter region. However, no mutations were identified in the sequences of the CRMP4 promoter core region in metastatic prostate cancer or prostate cancer cell lines, but it was identified that the methylation of a CpG island within the promoter region of the CRMP4 gene was responsible for downregulation of CRMP4 expression (49). CRMP4 is an intracellular phosphoprotein involved in the rib-like actin bundles of lamellipodia and the functional regulation of the actin cytoskeleton in motile cells (50), which may be relevant for metastasis suppression.

The biological function of CRMP4 has also been reported in pancreatic cancer, by Hiroshima *et al* (51). Of all the CRMP family members (CRMP1-5), only CRMP4 expression was found to significantly increase in pancreatic cancer compared with the corresponding non-cancerous pancreas tissues. Knockdown of CRMP4 in prostate cancer cell lines expressing high levels of CRMP4 did not affect cell proliferation, but did reduce cell invasion *in vitro*. CRMP4 expression was identified to be higher in poorly differentiated adenocarcinomas when compared with other histological types. In addition, CRMP4 expression was found to correlate with severe venous invasion and liver metastasis; however, no such correlation was identified with other parameters, including lymph node metastasis, lymphatic invasion, intrapancreatic neural invasion, extrapancreatic plexus invasion, curability or recurrence rate. Therefore, CRMP4 is considered to be involved in liver metastasis via venous invasion in pancreatic cancer, which is the first step of metastasis. In addition, CRMP4-positive groups exhibited significantly poorer two-year overall survival and relapse-free survival when compared with the CRMP4-negative groups. In multivariate analyses, the authors identified venous invasion and elevated CRMP4 expression as independent prognostic factors for overall survival, whereas lymphatic invasion and elevated CRMP4 expression were identified as prognostic factors for relapse-free survival. These results indicate that, in pancreatic cancer, CRMP4 may present as a marker for metastasis and a predictor for prognosis.

Neuroblastoma is the most common type of extracranial solid tumor in children and is derived from neural crest precursor cells. Neuroblastoma may differentiate into benign tumors, such as ganglioneuroma and ganglioneuroblastoma, or regress spontaneously. Furthermore, certain types of neuroblastoma demonstrate resistance to chemotherapy and easily metastasize. In our previous study, CRMP4 mRNA expression was found to increase in neuroblastoma cell lines during retinoic acid-induced cell differentiation (52). In addition, recent studies detected an increase in CRMP4 protein levels following retinoic acid treatment (53,54). In neuroblastoma tissues, CRMP4 expression was difficult to detect in poorly differentiated neuroblastoma cells, whereas positive CRMP4 expression was found in cells undergoing neuronal differentiation in ganglioneuroblastoma and in fully differentiated ganglionic cells in ganglioneuroma. Furthermore, in ganglioneuroblastoma and ganglioneuroma, CRMP4 expression was higher in cells undergoing neuronal differentiation than in cells with more differentiated features. CRMP4 expression was not observed in the normal ganglion cells of the surrounding ganglia in contrast to the neoplastic neuronal cells (53). These observations in neuroblastic tumors are similar to the expression patterns of CRMP4 in nervous system development, which demonstrate a transient upregulation of CRMP4 expression during ongoing neuronal differentiation.

The opposite effects on CRMP4 expression in prostate and pancreatic cancer metastasis may reflect the difference in the predominant expression of CRMP4 splice isoforms. Previous studies have revealed that the 75-kDa CRMP4 long isoform (CRMP4b or TUC-4b) and its short counterpart (CRMP4a or TUC-4a), exhibit opposite functions in neurite outgrowth (55,56). In addition, Pan *et al* (40) demonstrated that LCRMP1 promotes invasion in small cell lung carcinoma, whereas this action is antagonized by the CRMP1 short isoform. Pan *et al* also demonstrated that the 22- to 72-amino acid sequence of the N-terminal is important in filopodia formation, and that fusion of this N-terminus with the core region of CRMP2 may promote filopodia formation in prostate cancer. The presence of a 75 kDa CRMP4b isoform was detected in the Capan-1 pancreatic cancer cell line, and it was determined that CRMP4b is 100% homologous with LCRMP1 in the 22- to 72-amino acid sequence of the N-terminal. Thus, CRMP4b may be involved in pancreatic cancer malignancy.

### CRMP5 and cancer

The involvement of CRMP5 in cancer was first identified when the CRMP5 autoimmune antibody was detected in the sera of patients with paraneoplastic neurological syndrome, usually associated with small cell lung cancer and, less frequently, thymoma (57). CRMP5 antibodies were detected predominantly in patients with non-invasive thymoma, which suggested an association between CRMP5 and benign disease. CRMP5 antibodies were not found to correlate with the prognosis of small cell lung cancer, however a trend towards the longer survival of CRMP5-negative patients was observed (58).

The biological role of CRMP5 in tumor tissues was first reported in thoracic tumors. Neuroendocrine lung tumors comprise a family of tumors with shared neuroendocrine differentiation features, but exhibit diverse clinical behaviors. High-grade neuroendocrine lung tumor patients have a higher risk of a poor prognosis and must receive multimodal treatment. In addition, these tumors can be misdiagnosed as undifferentiated NSCLCs, including poorly differentiated lung adenocarcinoma or squamous cell carcinoma (59). A previous cohort study of 164 thoracic tumor specimens revealed strong and extensive CRMP5 expression in 98.6% of high-grade neuroendocrine lung tumors, including small cell lung carcinomas and large cell neuroendocrine carcinomas (60). However, this extensive staining was highly specific and was not observed in any of the squamous cell carcinomas or adenocarcinomas, even those with poorly differentiated phenotypes. By contrast, the majority of low-grade neuroendocrine lung tumors were negative for CRMP5 staining. Furthermore, in lymph node metastases, the pattern of CRMP5 expression exactly matched that observed in the corresponding primitive biopsy specimen. However, lung metastases originating from other carcinomas did not exhibit CRMP5 expression. Although CRMP5 was highly expressed in patients with high-grade neuroendocrine tumors with short survival and extensive stage disease, the weak CRMP5 expression identified in low-grade neuroendocrine tumors had no prognostic value. Since CRMP5 was not expressed in the neuroendocrine cells or any other cells in the normal adult lung, the expression of CRMP5 in tumor tissues may present as a useful marker to aid the pathological diagnosis of high-grade neuroendocrine tumors.

Glial tumors are the most common types of primary brain malignancies. Glioblastoma multiforme (GBM; grade IV astrocytoma) accounts for 80% of malignant astrocytomas and is characterized by an extremely poor prognosis; 50% of patients succumb to the disease within one year of diagnosis (61). Gene expression profile analysis revealed a set of 70 genes that were more highly expressed in rapidly progressing tumors, which stratified GBMs into two groups that differed by more than four-fold in the median duration of survival. In the group exhibiting CRMP5 gene overexpression, patients had a life expectancy of less than half of that of patients with low CRMP5 expression (62). Brot *et al* also found that CRMP5 demonstrates cytoplasmic and nuclear localization in GBM tumors, however, CRMP5 only exhibits a cytosolic distribution under physiological conditions (63). Furthermore, the authors revealed a novel CRMP5 short isoform which is present in the nuclear compartment of tumor cells. This short isoform of CRMP5 is a product of CRMP5 cleavage at the C-terminal that results in the exposure of the nuclear localization signaling site for active translocation. Additionally, nuclear CRMP5 increases cell proliferation activity and the abrogation of nuclear translocation prevents this activity. Therefore, nuclear CRMP5 may be responsible for the proliferative characteristic of glioblastoma.

## 3. Conclusion and prospects

CRMPs are major phosphoproteins found in the developing and adult nervous system. The biological functions of CRMPs in axonal outgrowth and neuronal differentiation may provide CRMP-expressing tumor cells with the characteristics associated with tumor migration, invasion and differentiation. The present study summarized that different members of the CRMP family are involved in different tumors; CRMP1 and CRMP2 in lung cancer, CRMP2 in breast and colorectal cancer, CRMP4 in prostate cancer, pancreatic cancer and neuroblastoma, and CRMP5 in neuroendocrine lung cancer and glioblastoma. In these types of cancer, the CRMP family members are associated with diverse functions, including metastasis, progression and prognosis/diagnosis (Table II). Thus far, the role of CRMPs in cancer has rarely been reported.

Compared with the expression of total CRMPs, in certain cancers, the phosphorylated CRMP levels and nuclear localization of CRMPs may be more important. As shown in Fig. 1, the majority of signaling molecules exert their functions through the phosphorylation of CRMPs. Although CRMPs were originally identified as cytosolic proteins, in certain cancers, the nuclear localization of CRMPs has been detected and may be useful for predicting the clinical outcome. Further study is required to elucidate the exact functions of CRMPs, particularly with regard to phosphorylation and nuclear localization in cancers.

## Figures and Tables

**Table I:** Nomenclature of the CRMP family.

CRMP1	CRMP2	CRMP3	CRMP4	CRMP5
DPYSL1	DPYSL2	DPYSL4	DPYSL3	DPYSL5
Ulip3	Ulip2	Ulip4	Ulip1	Ulip6
DRP1	DRP2	DRP4	DRP3	DRP5
C22	TOAD-64		hUlip	CRAM

CRMP, collapsin response mediator protein; DPYSL1, dihydropyrimidinase-like protein; Ulip, uncoordinated 33-like protein; hUlip, human homolog of Ulip; DRP, dihydropyrimidinase -related protein; TOAD-64, turned on after division, 64 kDa protein; CRAM, cysteine-rich acidic transmembrane protein.

**Table II:** Expression and biological values of CRMPs in cancers.

CRMP and cancer type	Expression of CRMP in tumor tissues	Prediction of clinical outcome by CRMP	Ref.
CRMP1			
Lung cancer	Increased	Yes	26
LCRMP1			
Lung cancer	Decreased	Yes	40
CRMP2			
Colorectal cancer	Increased	No	43
Lung cancer	T-CRMP2, increased; nuclear P-CRMP2, increased	T-CRMP2, no; nuclear P-CRMP2, yes	44
Breast cancer	T-CRMP2, decreased; nuclear P-CRMP2, increased	T-CRMP2, no; nuclear P-CRMP2, yes	45
CRMP4			45
Prostate cancer	Decreased in metastatic cancers or during cancer progression	Yes	49
Pancreatic cancer	Increased	Yes	51
Neuroblastic tumor	Increased in differentiated tumors	Yes	53,54
CRMP5			
Neuroendocrine lung cancer	Increased in high-grade subtypes	Yes, in high-grade subtypes	60
Glioblastoma	T-CRMP5, increased; nuclear CRMP5, increased	Yes	62

CRMPs, collapsin response mediator proteins; LCRMP1, long-form CRMP1; T-CRMP, total CRMP; P-CRMP, phosphorylated CRMP.

## References

[1] Minturn JE, Fryer HJ, Geschwind DH, Hockfield S (1995). TOAD-64, a gene expressed early in neuronal differentiation in the rat, is related to unc-33, a *C. elegans* gene involved in axon outgrowth. J Neurosci.

[2] Hamajima N, Matsuda K, Sakata S, Tamaki N, Sasaki M, Nonaka M (1996). A novel gene family defined by human dihydropyrimidinase and three related proteins with differential tissue distribution. Gene.

[3] Byk T, Dobransky T, Cifuentes-Diaz C, Sobel A (1996). Identification and molecular characterization of Unc-33-like phosphoprotein (Ulip), a putative mammalian homolog of the axonal guidance-associated unc-33 gene product. J Neurosci.

[4] Quinn CC, Gray GE, Hockfield S (1999). A family of proteins implicated in axon guidance and outgrowth. J Neurobiol.

[5] Byk T, Ozon S, Sobel A (1998). The Ulip family phosphoproteins-common and specific properties. Eur J Biochem.

[6] Horiuchi M, El Far O, Betz H (2000). Ulip6, a novel unc-33 and dihydropyrimidinase related protein highly expressed in developing rat brain. FEBS Lett.

[7] Fukada M, Watakabe I, Yuasa-Kawada J (2000). Molecular characterization of CRMP5, a novel member of the collapsin response mediator protein family. J Biol Chem.

[8] Inatome R, Tsujimura T, Hitomi T (2000). Identification of CRAM, a novel unc-33 gene family protein that associates with CRMP3 and protein-tyrosine kinase(s) in the developing rat brain. J Biol Chem.

[9] Li W, Herman RK, Shaw JE (1992). Analysis of the *Caenorhabditis elegans* axonal guidance and outgrowth gene unc-33. Genetics.

[10] Quach TT, Duchemin AM, Rogemond V (2004). Involvement of collapsin response mediator proteins in the neurite extension induced by neurotrophins in dorsal root ganglion neurons. Mol Cell Neurosci.

[11] Uchida Y, Ohshima T, Sasaki Y (2005). Semaphorin3A signalling is mediated via sequential Cdk5 and GSK3beta phosphorylation of CRMP2: implication of common phosphorylating mechanism underlying axon guidance and Alzheimer’s disease. Genes Cells.

[12] Fukata Y, Itoh TJ, Kimura T (2002). CRMP-2 binds to tubulin heterodimers to promote microtubule assembly. Nat Cell Biol.

[13] Cole AR, Causeret F, Yadirgi G (2006). Distinct priming kinases contribute to differential regulation of collapsin response mediator proteins by glycogen synthase kinase-3 in vivo. J Biol Chem.

[14] Hotta A, Inatome R, Yuasa-Kawada J, Qin Q, Yamamura H, Yanagi S (2005). Critical role of collapsin response mediator protein-associated molecule CRAM for filopodia and growth cone development in neurons. Mol Biol Cell.

[15] Goshima Y, Nakamura F, Strittmatter P, Strittmatter SM (1995). Collapsin-induced growth cone collapse mediated by an intracellular protein related to UNC-33. Nature.

[16] Uchida Y, Goshima Y (2005). Molecular mechanism of axon guidance mediated by phosphorylation of CRMP2. Seikagaku.

[17] Yoshimura T, Kawano Y, Arimura N, Kawabata S, Kaibuchi K (2005). Molecular mechanisms of neuronal polarity. Nihon Shinkei Seishinhin Yakurigaku Zasshi.

[18] Lin PC, Chan PM, Hall C, Manser E (2011). Collapsin response mediator proteins (CRMPs) are a new class of microtubule-associated protein (MAP) that selectively interacts with assembled microtubules via a taxol-sensitive binding interaction. J Biol Chem.

[19] Yoshimura T, Arimura N, Kawano Y, Kawabata S, Wang S, Kaibuchi K (2006). Ras regulates neuronal polarity via the PI3-kinase/Akt/GSK-3beta/CRMP-2 pathway. Biochem Biophys Res Commun.

[20] Yoshimura T, Kawano Y, Arimura N, Kawabata S, Kikuchi A, Kaibuchi K (2005). GSK-3beta regulates phosphorylation of CRMP-2 and neuronal polarity. Cell.

[21] Lykissas MG, Batistatou AK, Charalabopoulos KA, Beris AE (2007). The role of neurotrophins in axonal growth, guidance, and regeneration. Curr Neurovasc Res.

[22] Moolenaar WH (2000). Development of our current understanding of bioactive lysophospholipids. Ann N Y Acad Sci.

[23] Chun J, Weiner JA, Fukushima N (2000). Neurobiology of receptor-mediated lysophospholipid signaling. From the first lysophospholipid receptor to roles in nervous system function and development. Ann N Y Acad Sci.

[24] Arimura N, Inagaki N, Chihara K (2000). Phosphorylation of collapsin response mediator protein-2 by Rho-kinase. Evidence for two separate signaling pathways for growth cone collapse. J Biol Chem.

[25] Patrakitkomjorn S, Kobayashi D, Morikawa T (2008). Neurofibromatosis type 1 (NF1) tumor suppressor, neurofibromin, regulates the neuronal differentiation of PC12 cells via its associating protein, CRMP-2. J Biol Chem.

[26] Shih JY, Yang SC, Hong TM (2001). Collapsin response mediator protein-1 and the invasion and metastasis of cancer cells. J Natl Cancer Inst.

[27] Chen JJ, Peck K, Hong TM (2001). Global analysis of gene expression in invasion by a lung cancer model. Cancer Res.

[28] Shih JY, Lee YC, Yang SC, Hong TM, Huang CY, Yang PC (2003). Collapsin response mediator protein-1: a novel invasion-suppressor gene. Clin Exp Metastasis.

[29] Wu CC, Lin JC, Yang SC (2008). Modulation of the expression of the invasion-suppressor CRMP-1 by cyclooxygenase-2 inhibition via reciprocal regulation of Sp1 and C/EBPalpha. Mol Cancer Ther.

[30] Khuri FR, Wu H, Lee JJ (2001). Cyclooxygenase-2 overexpression is a marker of poor prognosis in stage I non-small cell lung cancer. Clin Cancer Res.

[31] Riedl K, Krysan K, Põld M (2004). Multifaceted roles of cyclooxygenase-2 in lung cancer. Drug Resist Updat.

[32] Masferrer JL, Leahy KM, Koki AT (2000). Antiangiogenic and antitumor activities of cyclooxygenase-2 inhibitors. Cancer Res.

[33] Grösch S, Tegeder I, Niederberger E, Bräutigam L, Geisslinger G (2001). COX-2 independent induction of cell cycle arrest and apoptosis in colon cancer cells by the selective COX-2 inhibitor celecoxib. FASEB J.

[34] Yao M, Lam EC, Kelly CR, Zhou W, Wolfe MM (2004). Cyclooxygenase-2 selective inhibition with NS-398 suppresses proliferation and invasiveness and delays liver metastasis in colorectal cancer. Br J Cancer.

[35] Xie D, Nakachi K, Wang H, Elashoff R, Koeffler HP (2001). Elevated levels of connective tissue growth factor, WISP-1, and CYR61 in primary breast cancers associated with more advanced features. Cancer Res.

[36] Wenger C, Ellenrieder V, Alber B, Lacher U (1999). Expression and differential regulation of connective tissue growth factor in pancreatic cancer cells. Oncogene.

[37] Kubo M, Kikuchi K, Nashiro K (1998). Expression of fibrogenic cytokines in desmoplastic malignant melanoma. Br J Dermatol.

[38] Shakunaga T, Ozaki T, Ohara N (2000). Expression of connective tissue growth factor in cartilaginous tumors. Cancer.

[39] Chang CC, Shih JY, Jeng YM (2004). Connective tissue growth factor and its role in lung adenocarcinoma invasion and metastasis. J Natl Cancer Inst.

[40] Pan SH, Chao YC, Hung PF (2011). The ability of LCRMP-1 to promote cancer invasion by enhancing filopodia formation is antagonized by CRMP-1. J Clin Invest.

[41] Pan SH, Chao YC, Chen HY (2010). Long form collapsin response mediator protein-1 (LCRMP-1) expression is associated with clinical outcome and lymph node metastasis in non-small cell lung cancer patients. Lung Cancer.

[42] Wang WL, Hong TM, Chang YL, Wu CT, Pan SH, Yang PC (2012). Phosphorylation of LCRMP-1 by GSK3beta promotes filopodia formation, migration and invasion abilities in lung cancer cells. PLoS One.

[43] Wu CC, Chen HC, Chen SJ (2008). Identification of collapsin response mediator protein-2 as a potential marker of colorectal carcinoma by comparative analysis of cancer cell secretomes. Proteomics.

[44] Oliemuller E, Peláez R, Garasa S (2013). Phosphorylated tubulin adaptor protein CRMP-2 as prognostic marker and candidate therapeutic target for NSCLC. Int J Cancer.

[45] Shimada K, Ishikawa T, Nakamura F (2013). Collapsin response mediator protein 2 is involved in regulating breast cancer progression. Breast Cancer.

[46] Goodyear S, Sharma MC (2007). Roscovitine regulates invasive breast cancer cell (MDA-MB231) proliferation and survival through cell cycle regulatory protein cdk5. Exp Mol Pathol.

[47] Upadhyay AK, Ajay AK, Singh S, Bhat MK (2008). Cell cycle regulatory protein 5 (Cdk5) is a novel downstream target of ERK in carboplatin induced death of breast cancer cells. Curr Cancer Drug Targets.

[48] Prasad CP, Rath G, Mathur S, Bhatnagar D, Parshad R, Ralhan R (2009). Expression analysis of E-cadherin, Slug and GSK3beta in invasive ductal carcinoma of breast. BMC Cancer.

[49] Gao X, Pang J, Li LY (2010). Expression profiling identifies new function of collapsin response mediator protein 4 as a metastasis-suppressor in prostate cancer. Oncogene.

[50] Rosslenbroich V, Dai L, Baader SL, Noegel AA, Gieselmann V, Kappler J (2005). Collapsin response mediator protein-4 regulates F-actin bundling. Exp Cell Res.

[51] Hiroshima Y, Nakamura F, Miyamoto H (2013). Collapsin response mediator protein 4 expression is associated with liver metastasis and poor survival in pancreatic cancer. Ann Surg Oncol.

[52] Gaetano C, Matsuo T, Thiele CJ (1997). Identification and characterization of a retinoic acid-regulated human homologue of the unc-33-like phosphoprotein gene (hUlip) from neuroblastoma cells. J Biol Chem.

[53] Choi YL, Kim CJ, Matsuo T (2005). HUlip, a human homologue of unc-33-like phosphoprotein of *Caenorhabditis elegans*; Immunohistochemical localization in the developing human brain and patterns of expression in nervous system tumors. J Neurooncol.

[54] Tan F, Wahdan-Alaswad R, Yan S, Thiele CJ, Li Z (2013). Dihydropyrimidinase-like protein 3 expression is negatively regulated by MYCN and associated with clinical outcome in neuroblastoma. Cancer Sci.

[55] Quinn CC, Chen E, Kinjo TG (2003). TUC-4b, a novel TUC family variant, regulates neurite outgrowth and associates with vesicles in the growth cone. J Neurosci.

[56] Yuasa-Kawada J, Suzuki R, Kano F, Ohkawara T, Murata M, Noda M (2003). Axonal morphogenesis controlled by antagonistic roles of two CRMP subtypes in microtubule organization. Eur J Neurosci.

[57] Yu Z, Kryzer TJ, Griesmann GE, Kim K, Benarroch EE, Lennon VA (2001). CRMP-5 neuronal autoantibody: marker of lung cancer and thymoma-related autoimmunity. Ann Neurol.

[58] Monstad SE, Drivsholm L, Skeie GO, Aarseth JH, Vedeler CA (2008). CRMP5 antibodies in patients with small-cell lung cancer or thymoma. Cancer Immunol Immunother.

[59] Asamura H, Kameya T, Matsuno Y (2006). Neuroendocrine neoplasms of the lung: a prognostic spectrum. J Clin Oncol.

[60] Meyronet D, Massoma P, Thivolet F (2008). Extensive expression of collapsin response mediator protein 5 (CRMP5) is a specific marker of high-grade lung neuroendocrine carcinoma. Am J Surg Pathol.

[61] Lacroix M, Abi-Said D, Fourney DR (2001). A multivariate analysis of 416 patients with glioblastoma multiforme: prognosis, extent of resection, and survival. J Neurosurg.

[62] Liang Y, Diehn M, Watson N (2005). Gene expression profiling reveals molecularly and clinically distinct subtypes of glioblastoma multiforme. Proc Natl Acad Sci USA.

[63] Brot S, Rogemond V, Perrot V (2010). CRMP5 interacts with tubulin to inhibit neurite outgrowth, thereby modulating the function of CRMP2. J Neurosci.

